# Urolithiasis secondary to primary obstructive megaureter in an adult: a case report

**DOI:** 10.1186/s13256-017-1342-z

**Published:** 2017-07-01

**Authors:** Somuah Tenkorang, Jean-Paul Omana, Soufiane Mellas, Fadl Mohammed Tazi, Jalal Eddine El Ammari, Abdelhak Khallouk, Mohammed Jamal El Fassi, Moulay Hassan Farih

**Affiliations:** Department of urology, Hassan II University Hospital, Fez, Morocco

**Keywords:** Primary obstructed megaureter, Urolithiasis, Adult, Hydroureteronephrosis, Stone

## Abstract

**Background:**

Primary obstructive megaureter is relatively uncommon in adults. This condition usually regresses spontaneously or is treated during infancy. It can remain asymptomatic for decades until adulthood when symptoms may manifest in the event of complications or during a routine radiologic imaging. Primary obstructive megaureter has been reported to favor stone formation in the upper urinary tract.

**Case presentation:**

We present the case of a 35-year-old Moroccan man who had a 10-year history of intermittent left flank pain and hematuria. A computed tomography urogram revealed a left megaureter with giant ureteral and renal calculi.

**Conclusions:**

Primary obstructive megaureter should be a differential diagnosis in an adult with hydroureteronephrosis associated with urolithiasis especially when there is kidney impairment. Through this case report we will discuss the diagnosis and management of primary obstructive megaureter associated with urolithiasis in adults.

## Background

Although primary obstructive megaureter (POM) is common in children, it is relatively uncommon in adults [1]. This can be explained by the fact that POM in children regresses spontaneously with time in most cases. Surgery is only indicated in complicated cases [2]. A relatively few cases of POM may not regress and may persist for decades until clinical manifestations occur in the event of complications such as urolithiasis, renal insufficiency, recurrent urinary infections, and pain [2, 3]. Urolithiasis secondary to POM was recently reported in the English literature. Our case is of great interest as it shows that POM associated with urolithiasis can be easily mistaken for hydroureteronephrosis secondary to urolithiasis in adults. Thus, POM should be ruled out as an underlying cause for urolithiasis associated with megaureter in adults. Furthermore, this case seeks to discuss how to best diagnose and manage this condition.

## Case presentation

A 35-year-old Moroccan man presented with intermittent left flank pain and macroscopic hematuria of 10-year duration. He had no relevant medical history apart from passing out stones on two occasions. He had taken only ibuprofen occasionally to relieve his pain.

A physical examination revealed that he was in a very good general condition. He was afebrile. His blood pressure was 120/80 mmHg. He had a slight left flank pain with no palpable mass. His body mass index (BMI) was 27 kg/m^2^. No other clinical signs were found after a complete physical examination.

A blood test revealed the following data: hemoglobin (Hb) 13.4 g/dl, white blood cells (WBC) 7500/mcL, C-reactive protein (CRP) 20 mg/L, blood urea nitrogen (BUN) 0.36 g/L, creatinine 11 mg/L, sodium 140 mEq/L, and potassium 3.8 mEq/L.

**Fig. 1:**
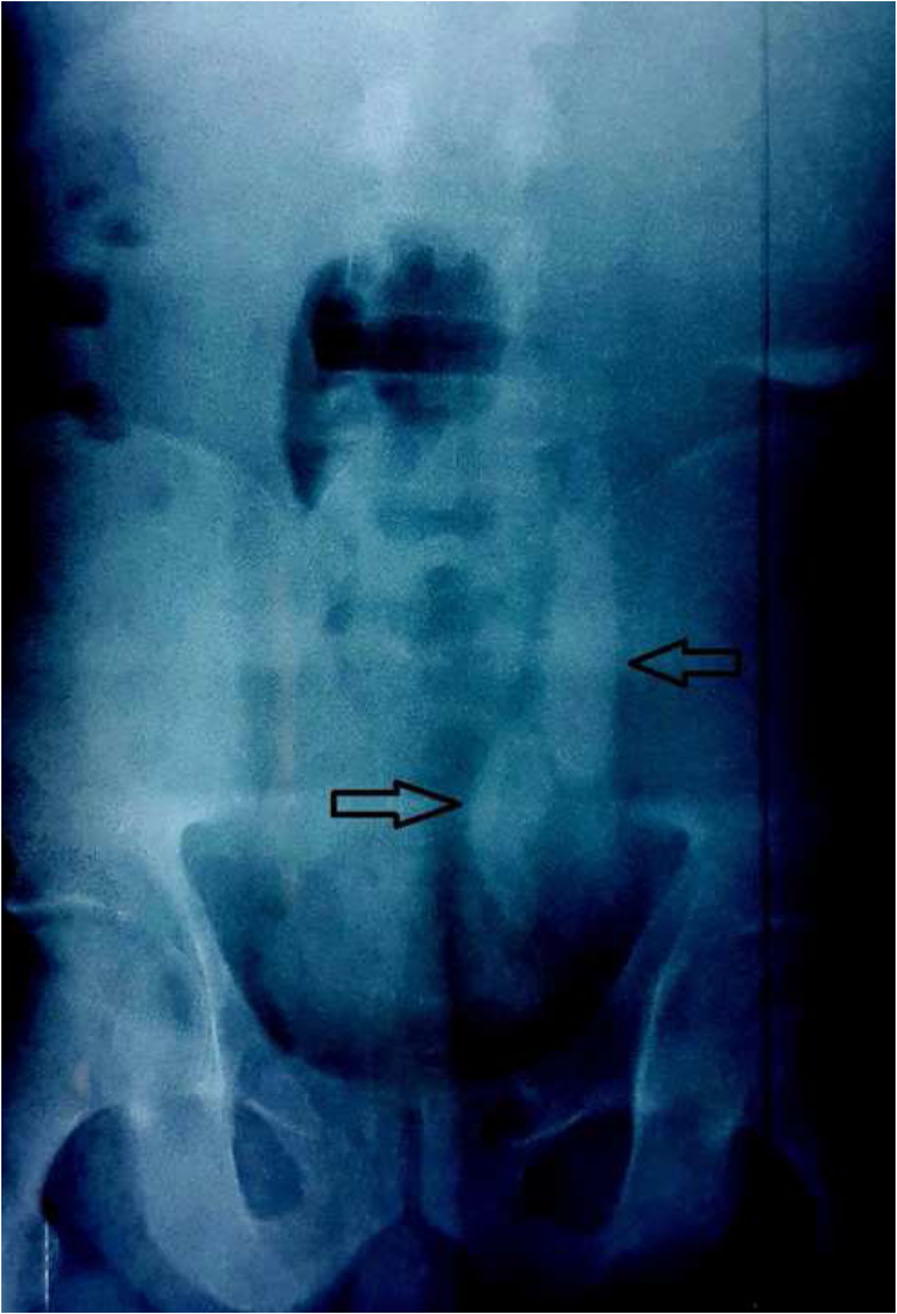
Kidney, ureter, and bladder X-ray showing 2 giant left ureteral stones. The *arrows* point to the two radio opaque left ureteral stones

Urine analysis revealed the following data: pH 5, glucose negative, proteins negative, leukocytes 320,000/ml, red blood cells (RBC) 10,000/ml, and crystals negative. A urine culture tested positive; it grew *Proteus mirabilis*. A kidney, ureter, and bladder (KUB) X-ray showed two giant ureteral stones and a left renal stone (Fig. 1).

**Fig. 2:**
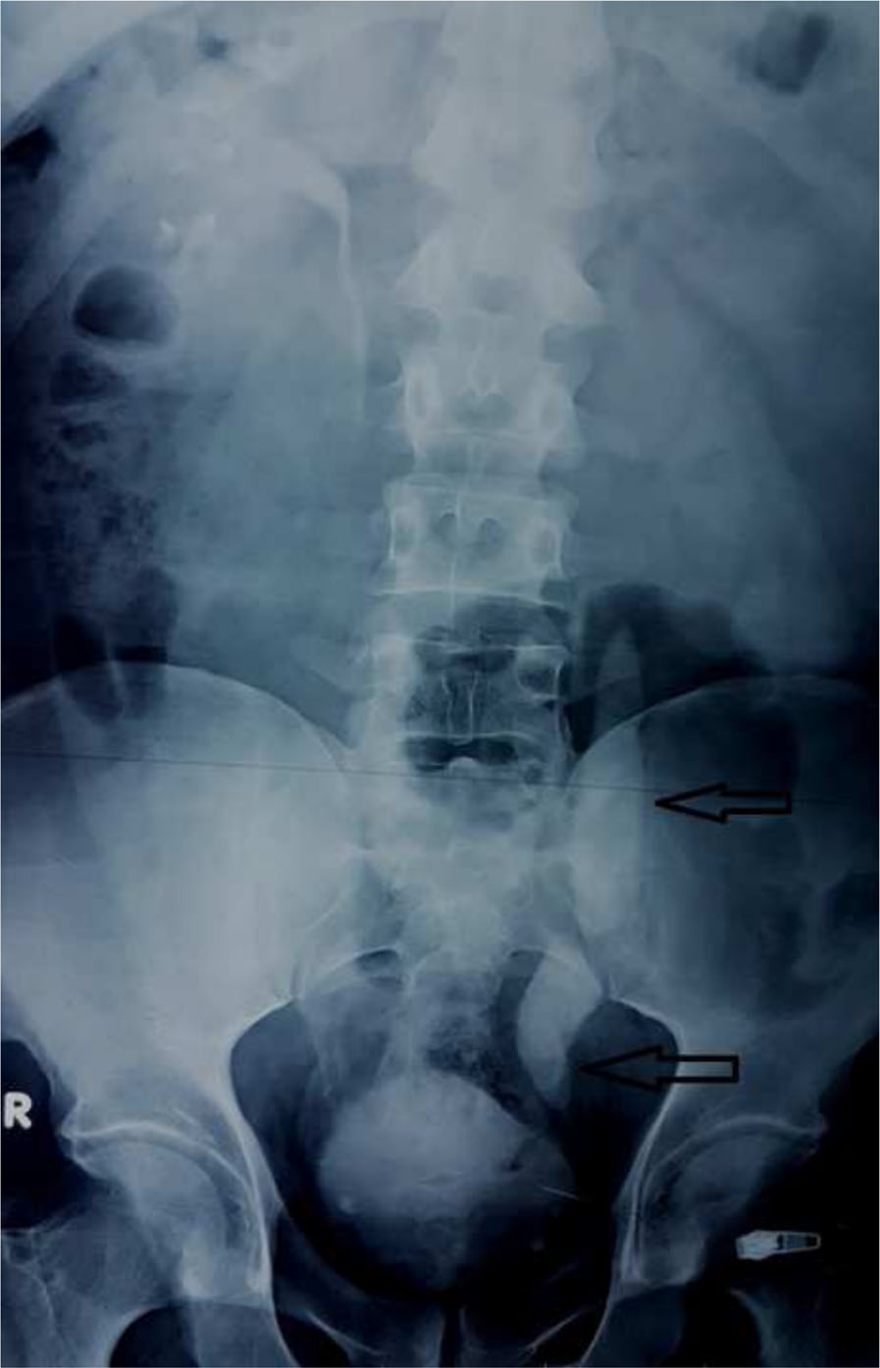
Intravenous pyelogram revealing no concentration and excretion at 1 hour from the left kidney consistent with a nonfunctioning left kidney. The *arrows* point to the two giant ureteral calculi

Abdominal ultrasonography (US) showed severe hydroureteronephrosis. An intravenous pyelogram (IVP) showed no concentration and excretion at 1 hour from his left kidney consistent with a nonfunctioning left kidney (Fig. 2).

**Fig. 3:**
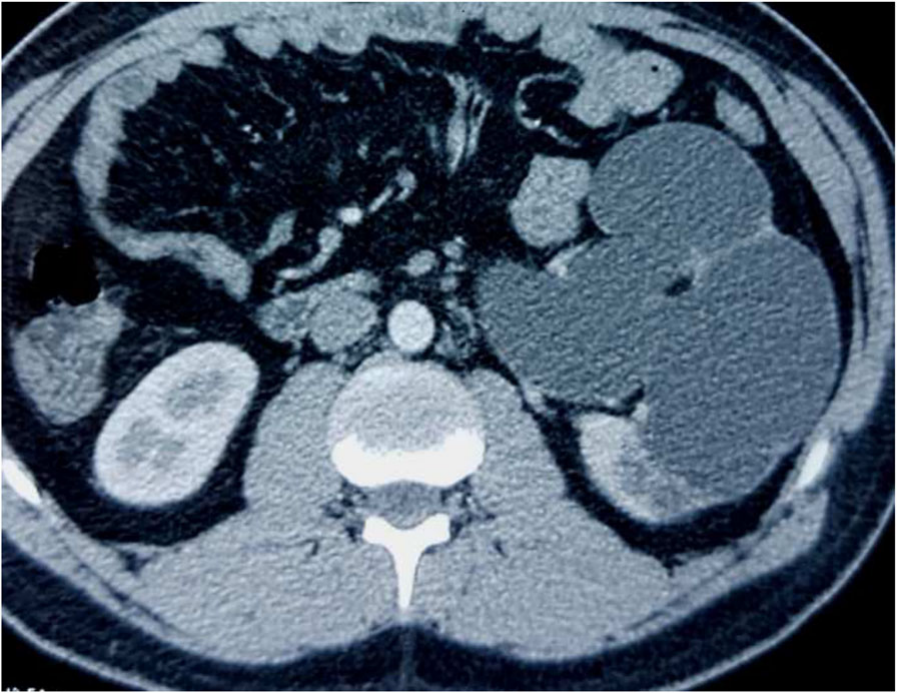
Axial enhanced computed tomography urography showing severe left hydronephrosis

**Fig. 4:**
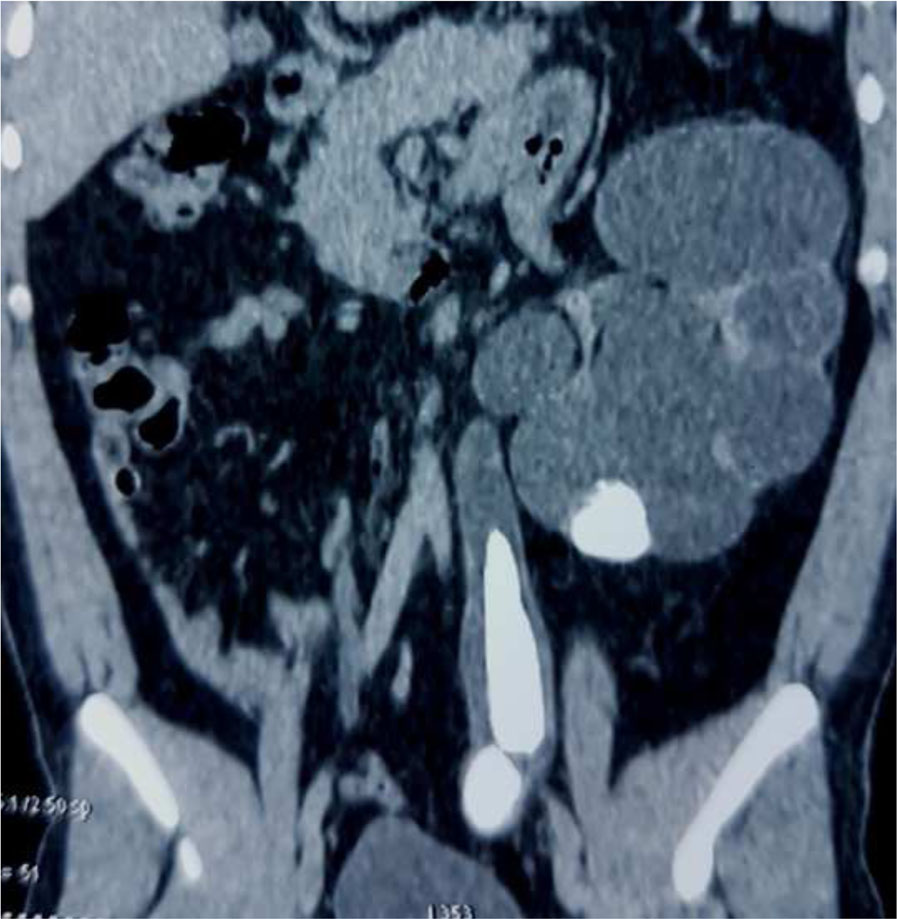
Coronal enhanced computed tomography urography showing severe hydroureteronephrosis associated with two giant left ureteral stones and a left inferior calyceal stone

Computed tomography urography objectified an advanced hydroureteronephrosis associated with two giant stones in the abdominal and pelvic ureter respectively and another stone in the left inferior renal calyx (Figs. 3 and 4). The ureteric stones measured 7×3 cm and 3×3.5 cm respectively. These stones had densities between 780 and 850 Hounsfield units (HU).

We established the diagnosis of nonfunctioning left kidney secondary to urolithiasis. Surgery was indicated. Our patient had a left nephroureterectomy. The distal segment of the ureter close to the ureterovesical junction appeared narrow suggestive of POM. Histopathologic examinations of the specimen confirmed our suspicion.

Gross examination of the specimen (left nephroureterectomy): the left kidney measured 13×7×4 cm; the left ureter appeared dilated and measured 18×2.5 cm containing multiple stones. A cut section of the left kidney showed multiple cystic lesions in the renal parenchyma. Microscopic examination of the specimen: the renal parenchyma showed fibrosis of the glomerular, a pseudo-thyroid appearance of the tubules, and interstitial lymphoplasmacytic infiltrate. There were metaplastic changes in the urothelium of the ureter with no dysplasia. No lesions of malignancy were found.

His postoperative course was unremarkable. He had no complaints after 6 months of follow-up. His renal function is stable (BUN 0.45 g/L, creatinine 10.5 mg/L).

## Discussion

Our case presents a megaureter associated with ipsilateral renal and ureteral calculi. The underlying cause which is POM could only be established during preoperative observation aided by histologic examination of the surgical specimen. It is less helpful to use imaging to establish the diagnosis of POM when there is kidney impairment as it is impossible to explore the diseased ureter via IVP.

There is no definition for megaureter in adults. However, the British Association of Pediatric Urologists (BAPU) defined a ureteric diameter over 7 mm as abnormal [1]. POM is characterized by a muscular structure alteration of the lower distal ureter close to the ureterovesical junction. This affected segment of the ureter becomes adynamic constituting a functional obstruction.

POM is relatively common in children with an increasing incidence since the advent of fetal US. In adults, this condition is quite uncommon and only occurs after decades of remaining asymptomatic without any spontaneous regression. Symptoms occur eventually in the event of complications such as recurrent urinary infection, recurrent flank pain, urolithiasis, and kidney function impairment [3, 4].

Diagnosis of POM is greatly dependent on radiological imaging. The criteria for diagnosis of POM includes dilated ureter, lower end of ureter ending in a smooth taper, absence of vesicoureteral reflux (VUR), absence of bladder outlet obstruction, and absence of secondary causes of lower ureteral obstruction [4–6].

The presence of calculi in the upper urinary tract associated with a nonfunctional kidney makes it challenging to easily establish the diagnosis of POM in an adult. It can be mistaken for hydroureteronephrosis secondary to ureteral stone obstruction. The reason being that POM associated with urolithiasis is uncommon in adults. In addition, it has clinical and radiological features similar to obstructive urolithiasis especially when there is a nonfunctional kidney.

Urolithiasis secondary to POM can be located in any dilated segment of the ureter as well as in the dilated renal calyces. The stones can either be in the ureter or in the renal pelvis and calyces as reports have shown in the literature [3, 7, 8]. In our case, the patient had both ureteral and renal calculi. The stasis of urine is a predisposing if not a causative factor for formation of stones [6].

POM is predominant in males and is often unilateral affecting the left ureter [9, 10].

IVP and CT urography are the most promising imaging modalities that can ascertain the diagnosis of POM when urolithiasis is involved. They show an entire or partly distended ureter with narrowing of its lower distal end at the ureterovesical junction. They show the presence of stones in the distended ureter. Stones may be present in the distended renal pelvis and calyces as well. When the affected kidney is nonfunctional, IVP and CT urography cannot reveal the obstructed ureter that will help establish the diagnosis for POM as in our case. Therefore, the diagnosis may go unnoticed or be suspected during surgery when a narrowed lower end of the ureter is observed as in our case. Histopathologic examination of the surgical specimen can reaffirm POM as the underlying cause as observed in our case.

A suspicion of POM after IVP or CT urography indicates that the following minimum investigations be conducted: (1) voiding cystourethrogram to rule out VUR and bladder outlet obstruction [11, 12]; and (2) urethrocystoscopy coupled with ureteroscopy to eliminate secondary causes of ureter obstruction [7].

Retrograde or antegrade ureteropyelograms are helpful tools to confirm the diagnosis of POM in cases where the kidney is nonfunctional.

Histologic findings on the nonfunctional distal end of the ureter can reveal abnormalities suggestive of POM [13].

Urolithiasis associated with POM in the adult requires surgery [2]. The goal is to treat any present infections, relieve obstruction by extracting the stones, and repair the obstructed ureter. Antibiotics are administered based on urine culture. The stones are treated depending on their size, morphology, and location. POM is repaired by resecting the adynamic distal end of the ureter and reimplantation with or without ureteral tailoring (ureteroneocystostomy). Nephroureterectomy is required when there is complete renal impairment [14, 15].

## Conclusions

POM complicated by urolithiasis in an adult can be easily mistaken for ureterohydronephrosis secondary to urolithiasis as these conditions have similar clinical manifestations and radiological imaging cannot easily differentiate between them, especially when there is renal function impairment. Radiological imaging appears to be a very useful tool to differentiate between these conditions and to avoid any errors in diagnosis.

IVP and CT urography are very important diagnostic tools to identify a POM. Voiding cystogram and cystoscopy are required to rule out bladder outlet obstruction and any secondary causes of obstructed ureter in order to confirm the diagnosis of POM.

Urolithiasis associated with POM in adults requires surgery in order to avoid renal function impairment and other complications.

## Abbreviations

BAPU: British Association of Pediatric Urologists
BMI: Body mass index
BUN: Blood urea nitrogen
CRP: C-reactive protein
CT: Computed tomography
HB: Hemoglobin
HU: Hounsfield unit
IVP: Intravenous pyelogram
KUB: Kidney, ureter, and bladder
POM: Primary obstructive megaureter
RBC: Red blood cells
US: Ultrasonography
VUR: Vesicoureteral reflux
WBC: White blood cells
